# SARS-CoV-2 conjugate vaccine elicits robust immune responses that can protect against evolving variants.

**DOI:** 10.1016/j.vaccine.2025.126988

**Published:** 2025-04-30

**Authors:** Melanie Carroll, Heather B. Fox, Anh Tran, Gowri Chellappan, Leonardo V. Rojas, Geetha Karengil, Fataneh Karandish, John W. Langston, Brent M. Fall, Mary M. Whalen, Michael J. McCluskie, Yves Durocher, Anup Datta, Subhash V. Kapre, Ivan A. Olave

**Affiliations:** a Viral Vaccines QC, Inventprise, Inc., Redmond, WA, USA; b Viral Vaccines R&D, Inventprise, Inc., Redmond, WA, USA; c Infectious Diseases, Human Health Therapeutics Research Centre, National Research Council Canada, Ottawa, ON, Canada; d Bacterial Vaccines R&D, Inventprise, Inc., Redmond, WA, USA; e Human Health Therapeutics Research Centre, National Research Council Canada, Ottawa, ON, Canada; f Life Sciences - NRC Human Health Therapeutics Research Center, National Research Council Canada, Montréal, QC, Canada; g Founder and Chairman, Inventprise, Inc., Redmond, WA, USA

## Abstract

The SARS-CoV-2 pandemic necessitated effective vaccines that can endure antigenic mutations. Here we demonstrate highly immunogenic conjugate vaccines that elicit broad cross-neutralization to variants of concern (VOC) in animal studies. By utilizing protein-protein conjugation and Toll-Like Receptor (TLR) agonist adjuvants we achieve enhanced immunogenicity compared to unconjugated equivalents. These vaccine candidates induced broad cross-protection against several VOC, a characteristic lacking in early COVID-19 vaccines. Murine neutralizing antibody (nAb) titers from animals vaccinated with Beta-only conjugates were equivalent between Beta, Delta, Omicron BA.1, BA.2, and BA.4/BA.5 variants, which were circulating up to three years after the antigenic Beta strain. Additionally, Beta-Delta bivalent conjugate vaccines readily prevented disease in hamster challenge. Together this demonstrates a vaccine with remarkably broad cross-protection and potential to protect for extended periods despite mutations, without requiring modified boosters or antigen adaption. These techniques can be applied to more recent SARS-CoV-2 strains, and other viruses, highlighting the benefits of protein-protein conjugation coupled with TLR agonist secondary adjuvants.

## Highlights

• SARS-CoV-2 spike protein conjugated to CRM-197 with TLR agonist adjuvants leads to high IgG and nAb.
• Beta-Only conjugates with TLR agonists induced broadly cross-reactive nAb in mice.
• Beta-only conjugate led to neutralization of variants that emerged 3 years after antigenic strain.
• Bivalent conjugate vaccines effectively protect hamsters from viral challenge.
• Protein conjugate platform applicable to any disease requiring enhanced immunogenicity.

## 1 Introduction

The global impact of COVID-19 was dramatically mitigated by rapid roll-out of effective vaccines. In their first year, vaccines reduced COVID-19 deaths by 63 % in 185 countries [1]. Although mRNA vaccines are generated swiftly [2], the breadth of protection offered by first generation mRNA vaccines waned rapidly [[3], [4], [5], [6], [7], [8], [9], [10], [11]], warranting frequent boosters to maintain adequate protection against disease [[3], [4], [5], [6], [7], [8], [9], [10], [11], [12]] COVID-19 is now endemic and continually evolving, so broadly protective vaccines that provide durable protection against continually evolving variants are still warranted.

The SARS-CoV-2 spike protein Receptor Binding Domain (RBD) interacts with Angiotensin Converting Enzyme 2 (ACE2) receptor on the host cell's surface, for cellular entry upon infection. The RBD is the primary binding site of neutralizing antibodies and the most appropriate antigen for vaccine development. This spike protein is metastable, existing in a prefusion state before binding the ACE2 receptor, then undergoing conformational transformation after binding [12]. Correct protein conformation for immunogenic recognition of the RBD and an appropriately targeted immune response is essential. Two-proline stabilized trimerized spike protein (S—2P) expressed in stable CHO cell pools was selected as the primary antigen for its immunogenicity, yields, rapid production, and stability profile [13]. Good Manufacturing Practice (GMP) stable CHO pools can be generated in 8 weeks with comparable quality to clonal cells. By using CHO pools, new and evasive pathogens can be addressed quickly, accelerating the approval pipeline. Accordingly, revision to guidelines to allow vaccine antigens derived from pooled cells in early clinical phases is warranted [14].

To maximize immunogenicity of S—2P, conjugation to rCRM197 carrier protein was evaluated. rCRM197 is a non-toxic mutant of diphtheria toxin, widely used in commercial vaccines to prevent infectious diseases and enhance immunogenicity [15]. Conjugation of bacterial polysaccharides to carrier proteins potently enhances immune responses to weakly immunogenic antigens [[16], [17], [18], [19], [20], [21]], especially in young children. Conjugate vaccines prime for memory responses, resulting in long-lived immunity in young and old alike [22]. Traditionally conjugation has been used to access T-cell mediated immunity in bacterial vaccines; however, recent advances demonstrate that protein-protein conjugation can also benefit viral subunit vaccines [[23], [24], [25], [26], [27], [28]].

To further improve S—2P immunogenicity secondary adjuvants were investigated. Leveraging Toll-Like Receptor (TLR) agonists has allowed vaccine manufacturers to enhance and target immune responses to the desired adaptive outcome [[29], [30], [31]]. In our proof-of-concept study, 3 M-052, a TLR-7/8 stimulant was tested for its ability to elevate IgG and neutralizing antibodies (nAb) compared to aluminum hydroxide (Al(OH)_3_) alone. To support rapid approval, TLR-9 agonist CpG 1018 was utilized in subsequent studies since it has similar downstream advantages and had been included in previously approved vaccines [32,33].

Following the proof-of-concept study, B.1.351 (Beta) and B.1.617.2 (Delta) variants were peaking worldwide, causing increased transmission, disease severity, mortality, and immune evasion [3,34]. Evidently these strains would lead to newer variants of unknown virulence, transmissibility, and immuno-escape, so Beta and Delta S—2P proteins conjugated to rCRM197 ± CpG 1018 were tested in mice and in hamster challenge studies. This work demonstrates that protein-protein conjugation and secondary adjuvants are useful methods to enhance immunogenicity of protein-subunit vaccines. Such techniques are suitable for improving existing and future pathogens beyond SARS-CoV-2.

## 2 Materials and methods

### 2.1 Growth and expression of Beta and Delta S—2P in CHO^2353^ cells

CHO^2353^ pooled cells were grown using BalanCD CHO Growth A media with supplements (ThermoFisher Scientific and Millipore-Sigma). Flasks were incubated at 37 °C, 5 % CO2, at 120 rpm, and grown via subculturing to VCD 5-10 × 10^6^ cells/mL. Protein expression was induced with 2 μg/mL Cumate (ARK Pharma), at 32 °C, 5 % CO2, at 120 rpm. Feed 4 and glucose were added continuously to maintain 35 mM glucose for 8 ± 1 days. Harvested cells were clarified via 0.2 μm tangential flow filtration (TFF) membrane (Sartorius). Clarified permeate received nuclease and protease inhibitors, glycerol, and was stored at −80 °C.

### 2.2 Protein purification

Beta S—2P protein was batch loaded onto NGL Covid-19 Spike Protein AR 2.0 Affinity resin (Repligen), using AKTA Avant 150 system (Cytiva Life Sciences) with a residence time of 4.9 min, then protein was washed and eluted (Supplementary Fig. 1). Concentration and diafiltration into PBS, pH, 7.4 was done at small-scale using 50 kDa or 100 kDa Amicon Ultra-15 spin-filters (Millipore-Sigma), and large scale using Discover 12 Green WaterSep mPES, 300 kDa, 1 mm, 51.8 cm^2^ hollow fiber TFF membrane (Sartorius). Concentrated S—2P protein was brought to 5 % sucrose, sterile filtered using a 0.2 μm Steriflip (Millipore-Sigma) and frozen at −80 °C. Concentration was determined with Pierce Modified Lowry Protein Assay using BSA standards (Thermo Fisher Scientific). Protein purity was evaluated by denaturation with 0.1 M DTT and Bolt 4–12 % Bis-Tris Mini Protein Gel SDS-PAGE gel systems with Coomassie blue staining.

### 2.3 Conjugation

EDC and s-NHS (Millipore-Sigma) dissolved in 0.1 M MES were added at specific ratios to rCRM197 protein 15 ± 2 mg/mL in 0.1 M MES, pH 6.5 ± 0.2 (Thermo Fisher Scientific), (Supplementary Table 1). After 7–10 min activation, NH2-PEG-COOH linker (Biochempeg) was added to rCRM197 and incubated for up to 1 h. Reaction was quenched with Tris buffer, pH 8.0 and dialyzed in 20 mM HEPES+0.5 M NaCl, pH 8.0 ± 0.2 at 4 °C. S—2P proteins in PBS (7 mg/mL) were adjusted to pH 5.1 ± 0.2. EDC and s-NHS was dissolved in 0.1 M MES pH 5.00 and pH 6.10 respectively, added to S—2P proteins and activated for 10 min at room temperature pH 5.2 ± 0.2. Derivatized rCRM197-PEG-COOH was added to activated S—2P proteins and incubated for 1 h at pH 6.6 ± 0.2. Conjugate was purified and buffer exchanged into PBS pH 7.2 using 100 kDa MWCO protein concentrators (Thermo Fisher Scientific).

### 2.4 Proof of concept mouse study formulation

Liposomal adjuvant was prepared using modified thin-film hydration method [35]. Lipid stocks of Dipalmitoylphosphatidylcholine (DPPC), cholesterol (Millipore-Sigma), and 3 M-052 adjuvant (3 M Drug Delivery Systems) were made in chloroform (Millipore-Sigma). The ratio of 3 M-052 adjuvant to lipids was 1:10 (*w*/w). Lipids were purged with N_2_ and chloroform evaporated. The resulting thin dried lipid film was hydrated in Water for Injection at 50 °C for 25 min. Liposomes were vortexed and sonicated at 50 °C for 15 min and stored at 2–8 °C until further use.

Final formulations consisted of 5 μg S-2P protein (Wuhan) and 12.5 μg aluminum hydroxide (Croda), 5 μg S-2P conjugate (Wuhan) and 12.5 μg aluminum hydroxide, or 5 μg S-2P conjugate (Wuhan) and 12.5 μg aluminum hydroxide and 0.2 μg 3 M-052.

### 2.5 Beta and Delta S—2P conjugate mouse study formulation

Beta S—2P and Delta S—2P (NRC Canada) spike protein rCRM197 conjugates were formulated at 5 μg per antigen with either 10 μg or 25 μg of CpG-1018 (Dynavax) and 375 μg aluminum hydroxide (Croda) or 750 μg aluminum hydroxide alone.

### 2.6 Beta and Delta S—2P conjugate hamster challenge study formulation

Beta S—2P and Delta S—2P spike conjugate formulations consisted of 5 μg of each Beta and Delta antigen rCRM197 conjugate plus either 100 μg of CpG-1018 and 350 μg aluminum hydroxide, or 750 μg aluminum hydroxide alone. Formulations were carried out per manufacturer instructions (Dynavax). Aluminum hydroxide concentrations were selected based on potential human doses.

### 2.7 Mouse studies

Mouse immunizations and sera collections occurred at Cocalico Biologicals. Animal work was approved by IACUC of Cocalico Biologicals, Inc. (Animal Welfare Assurance Number D16–00398 / A3669–01), and were compliant with ARRIVE guidelines. Sample sizes were estimates of suitable group sizes for statistically relevant data whilst adhering to 3Rs. Staff were blinded to the treatment groups.

### 2.8 Proof of concept mouse study

9-week-old female BALB/c mice (Charles River Laboratories) were acclimatized for 7 days and housed at 5 mice/cage. Animals were randomly assigned to experimental groups. All animals purchased were used in the study.

Intramuscular inoculations were administered to hind legs at days 0 and 14. Vaccine candidates tested are described in proof-of-concept mouse study formulation methods above, (*N* = 7/8 per group). Animals were monitored daily and found healthy throughout. Blood was collected on days 0, 7, 14 and 28, during which mice were anesthetized to surgical plane with Ketamine (2.5 mg)/ Xylazine (0.5 mg). Blood was collected via retro-orbital vein (Days 0, 7 and 14) or jugular sever (Day 28). At study termination, mice were anesthetized, exsanguinated, and subject to cervical dislocation.

### 2.9 Bivalent vs monovalent mouse study

7-week-old female BALB/c mice (Charles River Laboratories) were acclimatized for 2-weeks, and housed as described previously. Intramuscular inoculations were administered to hind legs at days 0 and 21. Sera was collected at Days 0, 21, 35 and 49. A subset of mice (3 per group) were exsanguinated at day 35 for splenectomies, reducing group numbers by 3 for day 49 analysis. Due to unforeseen circumstances, spleens were not viable for use in the study.

12 candidates were tested, consisting of 3 antigens each with 4 adjuvant formulations. Bivalent Beta and Delta conjugates (5 μg/conjugate/dose), Beta-only conjugate (5 μg/dose), and Delta-only conjugate (5 μg/dose), each with 4 adjuvant combinations: 1) 375 μg/dose aluminum hydroxide (Low Al(OH)3) (*n* = 6), 2) 750 μg/dose aluminum hydroxide (High Al(OH)3) (n = 6), 3) 375 μg/dose aluminum hydroxide +10 μg/dose (Low CpG) (*n* = 7), and 4) 375 μg/dose aluminum hydroxide +25 μg/dose (High CpG) (n = 7). PBS served as placebo (n = 7). 85 mice were used in total. Bleeds and exsanguinations were performed as described for proof-of-concept study.

### 2.10 Mouse studies: Binding IgG ELISAs

Sera was heat inactivated (HI) at 56 °C for 30 min. Immunoplates were coated with 1 μg/mL antigenic S—2P in PBS, blocked with 1 % BSA (Roche), washed, and incubated with 3-fold dilutions of HI-sera. Mouse IgG was detected with HRP-Goat-Anti-mouse IgG (Thermo Fisher Scientific). WHO Human Convalescent Sera (NIBSC) served as a reference standard in proof-of-concept study only. Human IgG was detected with HRP-Goat-Anti-human IgG (Thermo Fisher Scientific). Following washing 3,3′,5,5′-Tetramethylbenzidine (TMB) reagent (Thermo Fisher Scientific) was added and reaction stopped with 1 N HCl. Absorbance read at 450 nm - 570 nm on Biotek HTX plate reader (Agilent).

### 2.11 Mouse proof-of concept study: RBD-HRP neutralizing Ab Test

Serum neutralizing antibodies (nAb) were measured with SARS-CoV-2 Neutralization sVNT cPassTM Kit (GenScript) per manufacturer's instructions. At the time, pseudovirus neutralization assays were not established in our laboratory, so the RBD-HRP assay was utilized for rapid results.

### 2.12 Mouse studies: Pseudovirus neutralization assay

HEK-293T-hACE2 cells were maintained in Dulbecco's Modified Eagle's Medium containing 4 mM l-glutamine, 4500 mg per L glucose, 1 mM sodium pyruvate and 1500 mg per L sodium bicarbonate (DMEM High Glucose), supplemented with 10 % fetal bovine serum (FBS) and antibiotics (Thermo Fisher Scientific). Assay was adapted from published methods [36,37]. 2 × 10^4^ cells/well cells were seeded into white flat-bottomed 96-well plates (Thermo Fisher Scientific) and incubated overnight at 37 °C / 5 % CO_2._ HI-sera underwent 3-fold dilutions in DMEM from 1:20 to 1:43740. Commercially available pseudovirus particles containing SARS-CoV-2 VOC spike proteins and Luciferase reporters (Genecopoeia and BPS Bioscience) were diluted to 2 × 10^6^ TU/mL in DMEM High Glucose, mixed and incubated at 37 °C for 1-h. For virus-only controls, pseudovirus and media were mixed and incubated. Sera-pseudovirus samples were added to cells and incubated for 3 days at 37 °C / 5 % CO_2_. Bright-Glo Luciferase reagent (Promega) was added to the plates and Luciferase activity read (Agilent HTX). Half-maximal inhibitory concentration (IC50) analysis was performed with Graphpad Prism per published methods [36].

All mice sera was tested. Due to insufficient sample volumes, the following tests were performed with less than entire study group. Day 35 nAb: Omicron BA.2; Bivalent low Al(OH)_3_ *n* = 5, Delta high Al(OH)_3_ *n* = 5. Omicron BA.4/5: Bivalent low Al(OH)_3_ *n* = 1, Bivalent high Al(OH)_3_ *n* = 4, Bivalent low CpG *n* = 6, Bivalent high CpG n = 6, Beta-only low Al(OH)_3_ *n* = 3, Beta-only high Al(OH)_3_ n = 1, Beta-only low CpG *n* = 4. For day 49 nAb titer analysis, Omicron XBB.1.5 Bivalent low Al(OH)_3_ *n* = 2, Bivalent low CpG n = 3, Bivalent high CpG n = 3, Beta-only low Al(OH)_3_ n = 1, Beta-only high Al(OH)_3_ *n* = 0, Beta-only low CpG n = 2, and Beta-only.

### 2.13 Hamster Studies: Animals, viruses and study overview

7–8 week old (81–90 g) male Golden Syrian hamsters (Charles River Laboratories) were maintained at small animal facility of NRC Canada in accordance with the guidelines of Canadian Council on Animal Care. All procedures performed on animals in this study align with regulations and guidelines reviewed and approved in animal use protocol 2020.06 by the NRC Human Health Therapeutics Animal Care Committee. Treatment group sample sizes were determined by estimates of suitable numbers for statistically relevant data while adhering to 3Rs.

Hamsters were vaccinated intramuscularly via the tibialis anterior with Bivalent High Al(OH)_3_ (5 μg Beta-rCRM197 + 5 μg Delta-rCRM197 + 750 μg Al(OH)_3_ (*n* = 8)) and Bivalent Low Al(OH)_3_ CpG (5 μg Beta-rCRM197 + 5 μg Delta-rCRM197 + 350 μg Al(OH)_3_ + 100 μg CpG 1018 (*n* = 10)) or Placebo (PBS (n = 10)) at days 0 and 21 then challenged with Delta or Beta SARS-CoV-2 at Day 35. Animals were randomly assigned to treatment groups. Two additional hamsters were assigned to the naïve group and received no treatment. A total of 30 animals were utilized. Sera was collected from animals anesthetized using isoflurane inhalation on days 0, 21 and 35 via subclavian vein puncture. Terminal blood collection occurred immediately post euthanasia.

On day 35, hamsters were injected with Ketamine/Xylazine (90 kg/mg/8 kg/mg) and intranasally challenged with 1 × 10^5^ plaque forming unit (PFU) of virus or sterile PBS. Body weight was monitored daily. 5-days post-infection animals were euthanized by isofluorane inhalation, exposed to CO_2_, then necroscopy was performed. Lungs and nasal turbinate were harvested for plaque assay and viral genomic RNA (vgRNA) analysis. Lungs were harvested for histology and immunohistochemistry to the SARS-CoV-2 nucleocapsid. All infectious work was conducted under approved containment level-3 (CL-3) conditions at the NRC CL-3 facility.

SARS-CoV-2 Delta variant (hCoV-19/USA/MD-HP05647/2021; lineage B.1.617.2, BEI NR-55672), and Beta variant (hCoV-19/USA/MD-HP01542/2021; lineage B.1.351, BEI NR-55282) (BEI Resources, NIAID, NIH) were used for challenge. Viruses were propagated on Vero E6 cells and quantified on Vero cells. Sanger sequencing of spike gene confirmed exact genetic identity to original isolate. Passage 3 or 4 virus stocks were used in all subsequent experiments.

### 2.14 Hamster challenge study: Plaque assay

Virus burden was quantified by plaque assay at NRC CL-3 biocontainment facility as previously published [38]. Nasal turbinate and left lung were separately homogenized in PBS. Clarified homogenate supernatant were serially diluted 1:10 in infection media (DMEM, high glucose media supplemented with non-essential amino acid, 100 U/mL penicillin-streptomycin, 1 mM sodium pyruvate, and 0.1 % bovine serum albumin). Virus was adsorbed on Vero cells for 1-h at 37 °C, inoculum removed, and overlay media added (infection media with 0.6 % ultrapure, low-melting point agarose). Assay was incubated at 37 °C/5 % CO_2_ for 72-h. After incubation, cells were fixed with 10 % formaldehyde and stained with crystal violet. Plaques were enumerated and PFU determined per gram of tissue *n* = 8 animals/group.

### 2.15 Hamster challenge study: Plaque reduction neutralization tests (PRNT)

HI-serum was serially diluted 1-in-2 and incubated with equal volume of 100 PFU of Beta or Delta SARS-CoV-2 at 37 °C for 1-h. Viruses were adsorbed for 1 h at 37 °C on Vero cells, inoculum removed, then overlaid with media as described above. Assay was incubated at 37 °C/5 % CO_2_ for 72-h. Cells were fixed with 10 % formaldehyde and stained with crystal violet. No serum, virus-only back-titer control was included along with naïve animal serum. Placebo group *n* = 5, vaccine groups *n* = 8/group.

### 2.16 Hamster challenge study: Real time quantitative-PCR

Genomic viral RNA was quantified as previously described [40]. Viral genomic RNA from oral swabs were extracted using Quick-viral RNA kit per manufactures instruction (Zymo Research). Luna Universal One-step RT-qPCR kit was used to quantify viral genomic RNA (New England Biolabs) with primer/probe sets for the SARS-CoV-2 E gene [39] (Forward:5’ACAGGTACGTTAATAGTTAATAGCGT, Reverse:5’ATATTGCAGCAGTACGCACACA, Probe: ACACTAGCCATCCTTACTGCGCTTCG 5’Fam 3’QSY-1). Standards were generated with known concentrations of viral RNA copies. 5 μL of extracted RNA were run in duplicate on Applied Biosystems QuantStudio 3 (Thermo Fisher Scientific) and results analyzed with Design and Analysis Software DA2 version 2.6.0.

### 2.17 Hamster challenge study: RBD specific IgG ELISA

RBD-specific IgG ELISA was conducted with vaccinated hamster sera following previously published protocols [40]. Nunc MaxiSorp flat-bottom 96 well plates (Thermo Fisher Scientific) were coated with recombinant SARS-CoV-2 RBD-His recombinant protein (Sino Biological) and incubated overnight at 4 °C. Plates were washed with PBS / 0.1 % Tween-20 (PBS-T), blocked with 3 % bovine serum albumin (IgG-Free), then hamster serum was serially diluted 5-fold from 1:100 to 1:1562500, added to the plate and incubated for 1-h at 37 °C. Plates were washed with PBS-T then Peroxidase AffiniPure Goat Anti-Syrian Hamster IgG (H + L) (Jackson Immuno Research) added and incubated at 37 °C for 1-h. Following washes, Tetramethylbenzidine (TMB) substrate (Cell Signaling Technology) was added, incubated for 2-min at room temperature, then Stop solution (Cell Signaling) was added and absorbance measured at 450 nm. Inhibitory dilution 50 (ID50) was calculated using non-linear regression analysis. Analysis was performed for all animals in each study group.

### 2.18 Hamster challenge study: Histopathology and immunohistochemistry

Histology was performed by a CRO with scoring performed by a qualified pathologist (WaxIt). Four lobes of right lungs of infected hamsters were immersed in 10 % neutral buffered formalin for 1 week at room temperature. Fixed lungs were transferred to 70 % ethanol. Histopathology was performed on two animals from each challenge group, except Bivalent high Al(OH)_3_ group which did not undergo histopathology / immunohistochemistry. Samples were processed by standard paraffin embedding methods and blocks were cut into 5 μm thick sections, placed on glass slides, and subjected to hematoxylin and Eosin (H&E). Immunohistochemical (IHC) staining was completed at the NRC using modified protocol F on the Bond-Max III fully automated staining system (Leica Biosystems). All reagents from the Bond Polymer Refine Detection Kit were used. SARS-CoV-2 was detected using mouse anti-SARS-CoV-2 nucleocapsid monoclonal antibody (R&D Systems). Following deparaffinization and rehydration, sections were pre-treated with the Epitope Retrieval Solution 1 (ER1, Citrate buffer, pH 5.0) or Epitope Retrieval Solution 2 (ER2, EDTA buffer, pH 8.8) at 98 °C for 20-min. After washes, sections were quenched using peroxidase block for 5-min, washed again, then incubated for 15-min at room temperature with primary antibodies. Mouse-on-mouse superblock was applied for 15-min then anti-SARS-CoV-2 nucleocapsid antibody (PowerVision IHC/ISH Super Blocking, Leica Biosystems) was added. Sections were washed, incubated with polymer refine for 8-min at room temperature, developed with 3, 3′-diaminobenzidine (DAB) chromogen for 10-min then washed and counterstained for 6-min with hematoxylin, dehydrated, cleared and mounted. Negative controls included omission of primary antibody, secondary antibody alone, and lung tissue from naïve animals. IHC slides were scanned at 20× magnification using a Zeiss Axio Scan.Z1 digital slide scanner capable of brightfield imaging.

### 2.19 Statistics

All tests were performed as discrete tests, with results from each animal tested independently. All statistical analysis was performed using GraphPad Prism version 10.0.0 for Windows (GraphPad Prism Software). For the proof-of-concept mouse study, Mann-Whitney *t*-tests were performed to compare immune responses between each group. For serological and viral load comparisons non-parametric one-way ANOVA with multiple comparisons to placebo were performed. Gaussian distribution was not assumed. (Kruskal-Wallis test with Dunn's multiple comparisons test). All data are presented as Geometric Mean ± 95 % Confidence Interval (CI) unless otherwise stated. Statistical significance is denoted by asterisks in the figures (*P < 0.05, **P < 0.01, ***P < 0.001, and ****P < 0.0001), where alpha = 0.05.

## 3 Results

Beta and Delta S—2P expression in CHO^2353^ cells, purity, and characterization.

**Fig. 1:**
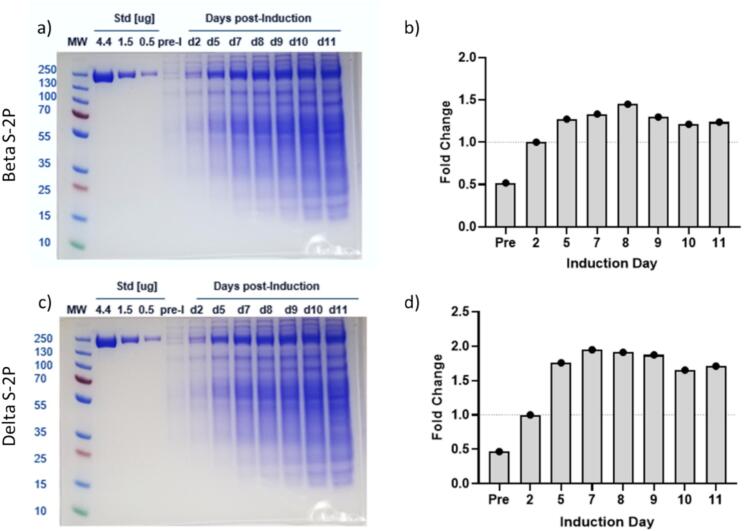
Induction time course of S—2P protein expression up to 11 days. CHO^2353^ pooled cells were grown using BalanCD CHO Growth A media supplemented with 50 μM MSX, 0.2 % KP188, and 0.1 % Anti-clumping solution. Flask was seeded with with 3 L of media at 3 × 10^5^ cells/mL and incubated at 37 °C, 5 % CO_2_, with 120 rpm, and grown via subculturing to a viable cell density (VCD) of 5–10 × 10^6^ cells/mL. After VCD was reached, protein expression was induced with 2 μg/mL Cumate, 125 μM MSX, and 5 % 0.8× Feed 4 at 32 °C, 5 % CO_2_, at 120 rpm. Feed 4 and glucose were added to maintain 35 mM Glucose for 8 ± 2 days of induction. Total growth time was 17–20 days from inoculation to harvest for 3 L culture. a) S—2P Beta SDS-PAGE gel showing change in expression over time of induction and b) Densitometry of Beta S—2P protein expression displaying pre-induction level and the fold change of Beta S—2P expression over course of induction, normalized to Induction Day 2 values, and indicated by the horizontal dotted line. c) S—2P Delta SDS-PAGE gel showing change in expression over time of induction and d) Densitometry of Delta S—2P protein expression displaying pre-induction level and the fold change of Delta S—2P expression over course of induction, normalized to Induction Day 2 values, indicated by the horizontal dotted line.

**Fig. 2:**
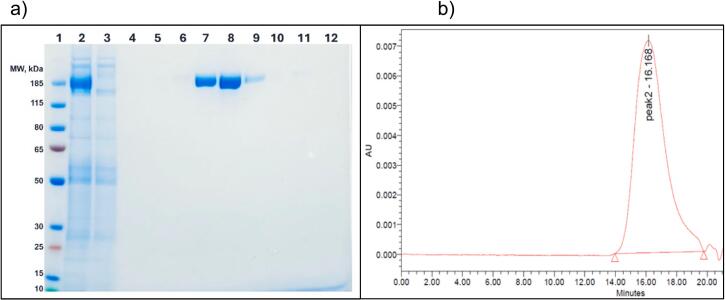
Representative S—2P Beta data as shown in a) SDS-PAGE gel of chromatography stained with Coomassie blue. Lane 1 is ladder; lane 2 is S—2P Beta protein column load; lane 3 is flow through material followed by a PBS wash in lane 4; lanes 5–12 are elution fractions with S—2P protein eluted in lanes 7–9. Protein was denatured with 0.1 M DTT and ran on Bolt 4–12 % Bis-Tris Mini Protein Gel SDS-PAGE gel system in MOPS buffer (Thermo Fisher Scientific), at 200 V for 42 min. Gel was stained with SimplyBlue Safe Stain (Thermo Fisher Scientific) for 60 min, and destained in water prior to imaging. b) SEC-HPLC profile of SARS-CoV-2 S-2P Beta proteins detected at 220 nm (red trace). Protein of interest has a retention time of 16.168 min and is labeled as peak2. Sample was run on Shodex 804 and 805 columns in series and eluted using 10 mM Potassium Phosphate buffer, pH 7.0, at 1.0 mL/min for a run time of 30 min. Buffer peaks at R_t_ > 21.00 min are excluded from the SEC-HPLC profile. (For interpretation of the references to colour in this figure legend, the reader is referred to the web version of this article.)

Purified S—2P proteins expressed in CHO cells were purchased from NRC Canada to test conjugation and formulation in animal studies. Corresponding stable CHO^2353^ pooled Beta and Delta S2—P expressing cells were acquired, and protein expression methods were adapted from NRC [[40], [41], [42], [43], [44]]. In a fed-batch cumate induction system, Beta and Delta S—2P protein expression stabilized after 8-days of induction (Fig. 1a and b). Chromatography and tangential flow filtration (TFF) was used to purify SARS-CoV-2 S-2P proteins. Experimental affinity chromatography resin for binding the RBD (Repligen) was used for purification. Maximum binding capacity was 4.1 mg S-2P/mL resin, lower than capacity for RBD alone, nevertheless an effective method to isolate S—2P protein. A representative chromatogram of Beta S—2P purification is shown in Supplementary Fig. 1, and correlates to fractions represented in the SDS-PAGE gel in Fig. 2a. Host cell protein and DNA were measured and both found to be <100 ppm (Supplementary Table 1). This reproducible method yielded 80 mg protein/L culture and > 95 % purity determined by SDS-PAGE and HPLC-SEC (Fig. 2b), providing high quality drug substance for conjugation.

### 3.1 Conjugation of Beta and Delta S—2P to rCRM197 carrier protein

**Fig. 3:**
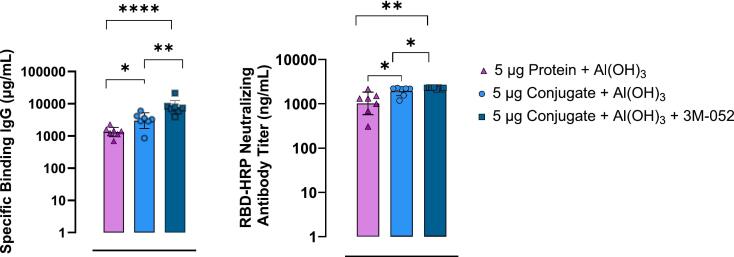
Dose, Conjugation and 3 M-052 improve immune responses in mice: Young female BALB/c mice (Protein + Al(OH)_3_*n* = 7, Conjugate + Al(OH)_3_ n = 7, Conjugate + Al(OH)_3_ + 3 M-052 *n* = 8) were inoculated intramuscularly with two doses of each vaccine candidate or placebo (PBS) 14 days apart. Mouse sera collected 2 weeks post-boost (Day 28), was tested for specific binding IgG to Wuhan S—2P (a) and RBD neutralizing antibodies (b). Data is displayed as Geometric Mean ± 95 % CI. Two-tailed Mann-Whitney tests were performed to determine statistical significance.

**Fig. 4:**
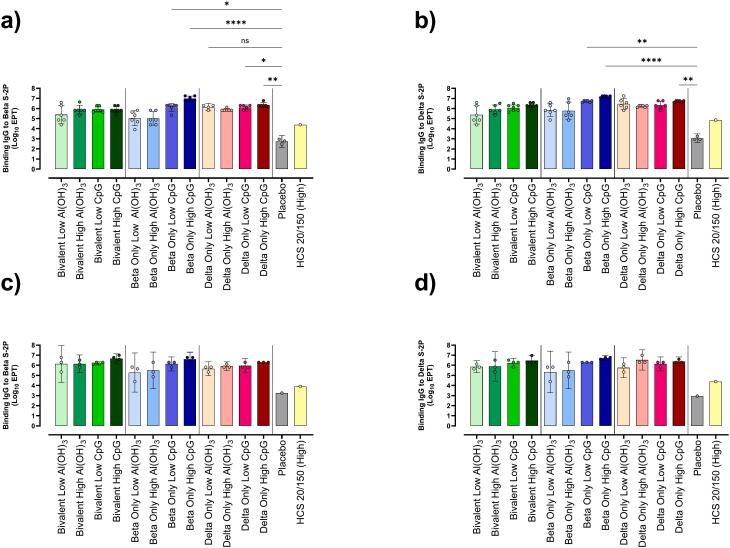
Binding IgG to Beta and Delta SARS-CoV-2 S-2P. Binding IgG endpoint titers were tested in mouse sera collected at day 35 (Beta a, Delta b) and day 49 (Beta c, Delta d). Endpoint titers are defined as the reciprocal of the highest detectable dilution above the cutoff value, where the cutoff is the background +3 SD of background. For day 35 analysis all bivalent groups and all groups with CpG 1018 contained 7 animals/group, while Beta-only and Delta-Only with only Al(OH)_3_ contained 6 animals/group. Day 49 data contains 4 animals/group for all bivalent groups and all CpG 1018 groups, while the Beta-only and Delta-Only with only Al(OH)_3_ had 3 animals/group. Data shown as Geometric Mean ± 95 % CI. One-way ANOVA with Dunn's multiple comparison to placebo was performed to determine statistical significance.

Process parameters affecting conjugation efficiency of S—2P protein with rCRM197 were identified as S—2P and carrier protein concentrations, stoichiometric ratios of the proteins, 1-Ethyl-3-(3-dimethylaminopropyl)carbodiimide hydrochloride (EDC), Sulfo N-hydroxysuccinimide (s-NHS) and rCRM197-PEG-COOH concentrations, and conjugation reaction duration. Supplementary Table 2 lists the reagent ratios and conditions used for conjugation of Beta and Delta S—2P with rCRM197. Fig. 2b and Supplementary Fig. 2 represent the size exclusion chromatography (SEC) profiles of purified Beta (molecular weight of the highest peak (M_p_) at R_t_ 16.168 min) and Delta proteins (M_p_ at R_t_ 15.938 min). Conjugate formation and conjugation efficiency were confirmed by comparing the SEC profiles of the conjugates to corresponding pure proteins (Supplementary Figs. 3a and 3b). Conjugate formation was confirmed based on the shift in R_t_ to lower values of 13.0 to 14.5 min compared to that of the pure protein peaks with R_t_ around 16.0 min (Fig. 3a and b). Conjugation process conditions were optimized to achieve >70 % conjugation efficiency based on the % unconjugated protein peak area around retention time (R_t_) 16.1 min and 15.9 min in Beta (Fig. 4a) and Delta (Fig. 4b) conjugate samples, respectively.

### 3.2 Conjugation and TLR-agonist improve immunogenicity in mice

To examine if conjugation and secondary adjuvants enhanced immune responses compared to aluminum hydroxide alone, an accelerated proof of concept study was performed. Animals vaccinated with Conjugate and Al(OH)_3_ had higher binding IgG compared to animals vaccinated with Protein and Al(OH)_3_ two-weeks post-boost. 3 M-052 further enhanced titers compared to conjugation alone. Specific binding IgG levels between protein + Al(OH)_3_ and conjugate + Al(OH)_3_ + 3 M-052 were significantly higher (*P* = 0.0006) (Fig. 3a). Similar results were observed for RBD-HRP Neutralizing Antibody titers, with conjugation and addition of 3 M-052 significantly improving RBD-HRP nAb titers (*P* = 0.0012) (Fig. 3b). Consequently, subsequent studies focused on S—2P conjugates with and without secondary adjuvants.

### 3.3 Bivalent vs monovalent vaccine immunogenicity in mice

As new virus variants continued emerging, a vaccine targeting more relevant VOC than Wuhan was imperative. Here, bivalent conjugate vaccines of Beta–rCRM197 + Delta-rCRM197 (Bivalent) were compared to monovalent conjugate vaccines Beta-rCRM197 (Beta only) or Delta-rCRM197 (Delta only) each in the presence of four adjuvant combinations. The TLR-9 agonist CpG 1018 was used as a secondary adjuvant to support a streamlined path to approval.

**Fig. 5:**
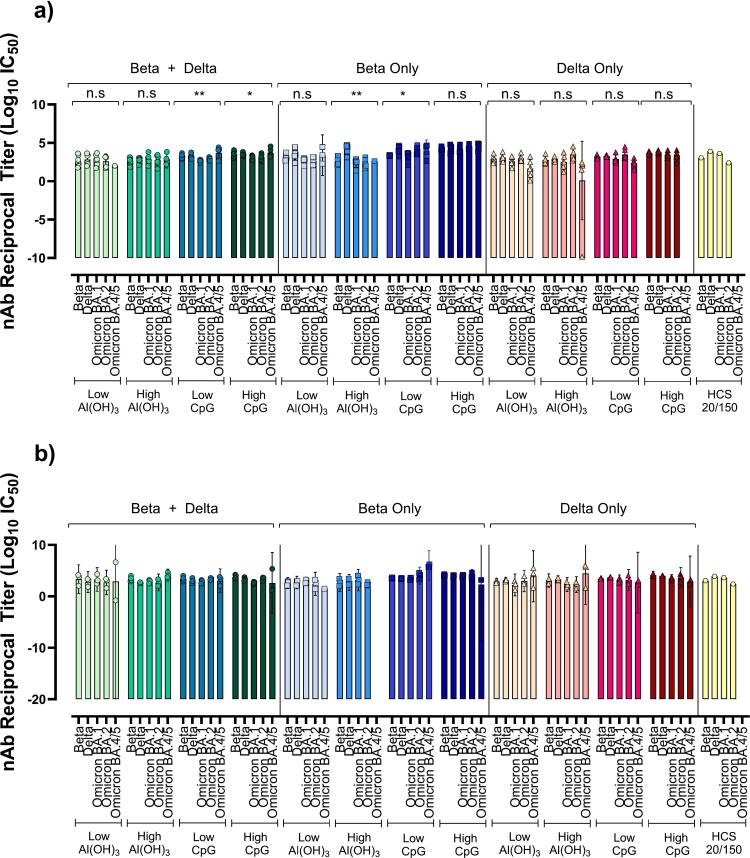
Neutralizing Antibody Titers to Variants of Concern in Day 35 and Day 49 Mouse Sera. Neutralizing antibody titers in mouse sera from day 35 (5a) and day 49 (5b). Data is presented as GMT ± 95 % CI. For day 35 analysis all bivalent groups and all groups with CpG 1018 contained 7 animals/group, while Beta-only and Delta-Only with only Al(OH)_3_ contained 6 animals/group. Day 49 data is obtained from 4 animals/group for bivalent and CpG 1018 groups, 3 animals/group for Beta-only and Delta-Only with only Al(OH)_3_. To determine if sera from the same group neutralized the SARS-CoV-2 variants differently, One-Way ANOVA with Dunn's Multiple Comparison was performed on results of each vaccine group.

Day 35 and day 49 sera was assessed for Binding IgG to Beta S—2P and Delta S—2P (Fig. 4), and nAb to Beta, Delta, Omicron BA.1, BA.2, BA.4/5, and XBB 1.5 (Fig. 5). As sera depleted, not all VOC could be included in all nAb tests. To maximize information obtained, nAb to Omicron BA.4/5 variants were measured in day 35 sera, and Omicron XBB 1.5 was measured in day 49 sera.

Regardless of immunogen, the Beta-only high CpG generated the highest binding IgG titers to both Beta and Delta S—2P at day 35 (*P* ≤0.0001) compared to placebo (Fig. 4). The titers of the Bivalent High CpG were equivalent to Beta-only High CpG groups by day 49; interestingly, this titer was reached two weeks earlier for the Beta-only High CpG candidate. The reason for this is unclear, however other groups show similar patterns for nAb in other beta-based vaccine studies [45].

All vaccine candidates elicited nAb capable of cross protection to variants tested, to varying degrees. There was no significant difference in titers obtained for each variant tested for Bivalent Low Al(OH)_3_, Bivalent High Al(OH)_3_, Beta-only Low CpG, Beta-only High CpG, and all Delta-only vaccines. The titers from Delta-only vaccines low across the board, so were excluded from further investigation. Surprisingly, the highest titers were from the Beta-only High CpG vaccine rather the Bivalent candidates, which were expected to be superior based on dual antigens and a higher overall dose. Statistical analysis could not be applied to day 49 serological data, as there were only 3–4 samples per group. This was due to half the mice in each group undergoing splenectomies on day 35. Due to circumstances beyond our control, spleens were unsuitable for use in cell mediated immunity experiments. Nevertheless, the data shows robust titers to all variants tested, possibly indicating protection against VOC that emerged long after the antigenic strains.

### 3.4 Variant timeline

**Fig. 6:**
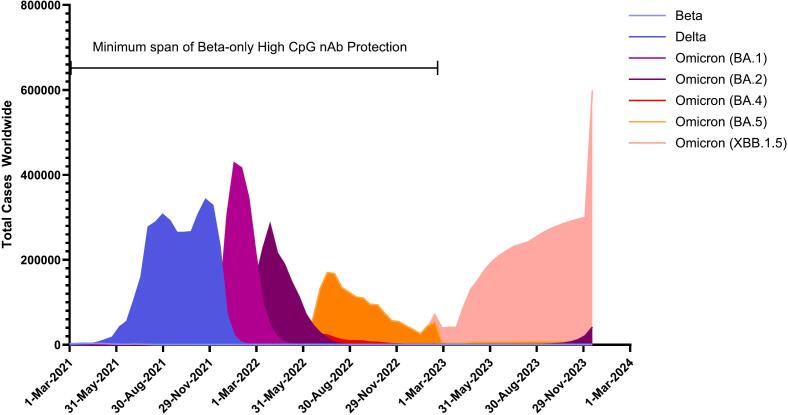
Timeline of SARS-CoV-2 Variant Cases. Worldwide case numbers of SARS-CoV-2 variants over time were obtained from https://ourworldindata.org/grapher/covid-variants-area sourced from GISAID, via CoVariants.org and plotted to assess theoretical longevity of conjugate vaccine candidates, based on equal murine nAb serological titers to VOC tested via pseudovirus neutralization.

To apply theoretical longevity to the protection against variants inferred by the mouse nAb data, a timeline of worldwide cases of each variant that was tested for murine nAb was generated using data from https://ourworldindata.org/grapher/covid-variants-area using data from GISAID via CoVariants.org [46] (Fig. 6). Beta variant emerged in August 2020 and Delta in October 2020. The nAbs produced from these vaccine candidates neutralized variants that were still in circulation three years after the antigenic strain emerged, at minimum.

### 3.5 Hamster challenge study

Serological data from mice studies indicated that all vaccines were likely to offer protection from disease, with Bivalent and Beta-only High CpG candidates the most promising. To confirm, efficacy study in hamsters was performed with the Bivalent vaccine candidate ± CpG 1018. Beta-only High CpG candidate was not included in the challenge study because the rapid evolution of the pandemic resulted in the challenge study being initiated prior to acquiring all the serological data in the previous mouse study, so the broadly cross-protective nAb titers were not yet discovered.

**Fig. 7:**
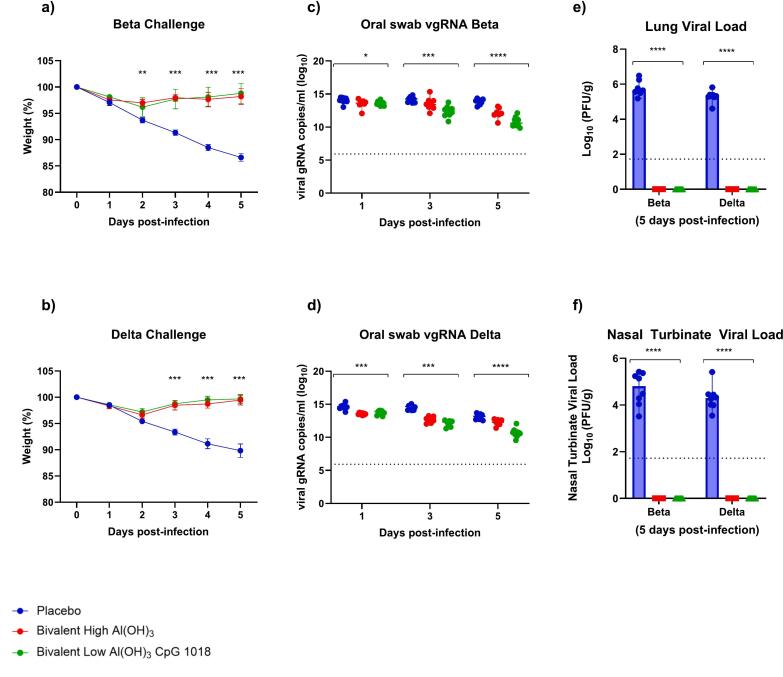
Bivalent SARS-CoV-2 vaccine candidate protected hamsters when challenged with Beta and Delta – Post-challenge body weight (a, b), viral genomic RNA from oral swabs for Beta variant (c) and Delta variant (d) and day 35 lung viral load and nasal turbinate viral load are shown in panels (e) and (f) respectively. Assay LOD represented by dashed lines. For body weight and oral swab vgRNA data, the placebo and bivalent low Al(OH)_3_ CpG 1018 groups contained 10 animals per group, while bivalent high Al(OH)_3_ group contained 8 animals. For viral load data, 8 animals per group were tested. Data was assessed by one-way ANOVA with Dunn's multiple comparisons to determine statistical significance. Body weight data is shown as SEM, vgRNA and Viral Load data is displayed as median ± 95 % CI. vgRNA results for Placebo vs Bivalent with low Al(OH)_3_ were as follows: Day 1 (Beta *P* = 0.0213, Delta *P* = 0.0094), day 3 (Beta *P* = 0.0002, Delta *P* = 0.0001) and day 5 (Beta *P* ≤0.0001, Delta P ≤0.0001).

Following challenge, body weights of vaccinated animals decreased to day 2, then returned to starting weight by day 5 post challenge (Fig. 7a and b). Animals in the placebo group continued losing weight to day 5 when study was terminated. The challenge variant did not impact the pattern seen in body weight. Additionally, no difference was observed between body weight responses in animals vaccinated with Bivalent High Al(OH)_3_ compared to Bivalent Low Al(OH)_3_ and CpG.

Relative to placebo, vaccinated animals showed significantly lower vgRNA levels in oral swabs taken on 1-, 3-, and 5-days post challenge (Fig. 7c, d). At 5-days post-challenge, no virus was detected in the lung or nasal turbinate when assessed by plaque assay, regardless of variant used for the viral challenge (Fig. 7e, f). Conversely, the placebo group exhibited high levels of live virus in the sampled tissues.

**Fig. 8:**
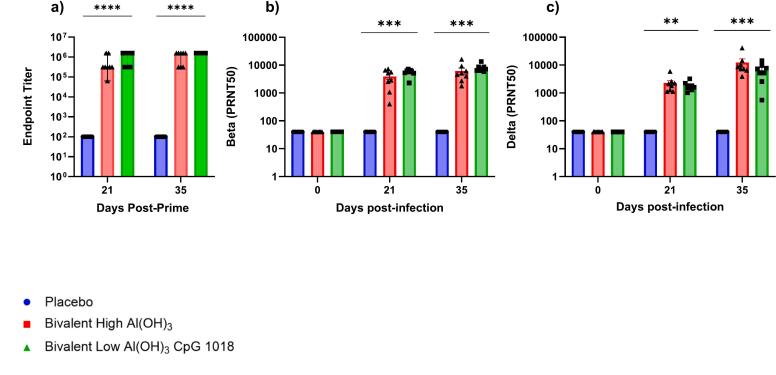
Hamster Serum RBD IgG and nAb. Hamster IgG levels to the SARS-CoV-2 RBD were assessed at days 21 and 35 by ELISA (8a) Endpoint titers were expressed as the reciprocals of the final detectable dilution with an OD above the cut-off value, which was defined as the average OD of the placebo. Placebo and Bivalent with CpG groups contained 10 animals each, while bivalent with Al(OH)_3_ contained 8 animals. For PRNT_50_ (8b, c), where PRNT_50_ is defined as the highest dilution of serum that results in 50 % reduction of plaque-forming units, sera from 5 animals from the placebo group were tested, and sera from 8 animals from each of the vaccine groups were tested. For all data in Fig. 8, One-Way ANOVA with Dunn's multiple comparison was carried out to determine significance.

Both vaccine candidates induced high levels of IgG after a single dose of vaccine. The IgG levels on day 21 were higher in the CpG adjuvanted group, however by day 35 the IgG levels were equivalent between groups. Similar results were observed for PRNT_50_ tests to Beta and Delta variants, with high levels of nAb to both variants seen by day 35 (Fig. 8).

Hematoxylin and Eosin (H&E) staining was performed on lung tissue collected at necroscopy from Naïve, Placebo and Bivalent Low Al(OH)_3_ and CpG groups. Pathology scores are indicated in Table 1. H&E staining was not performed on the Bivalent High Al(OH)_3_ group. Challenge with live virus in the Placebo group led to multiple lung pathologies, and overall cytopathic effect, with Delta leading to more severe pathologies than Beta. The vaccinated group showed only mild pathology, except for moderate Alveolar Histocytes for one animal challenged with Delta variant. Overall, no cytopathic effect was observed for vaccinated animals challenged with either Beta or Delta SARS-CoV-2, conversely animals in the Placebo group displayed clear cytopathic effect.

**Table 1:** Histopathology scores of infected vaccinated lung tissues. Scoring legend: Mild = 1, Moderate = 2 and Severe = 3.

Treatment	PBS Control		Placebo				Bivalent + Al(OH)_3_ + CpG			
Reference#	1	2	3	4	5	6	7	8	9	10
Challenge Strain	N/A		Beta		Delta		Beta		Delta	
Necrosis	0	0	0	0	0	0	0	0	0	0
Percentage Section Involved	0	0	25	50	70	70	1	1	5	10
Alveolar Hemorrhage	0	0	0	0	0	1	0	0	0	0
Airway Inflammation	0	0	1	2	2	3	0	0	0	0
Alveolar Edema	0	0	2	2	2	2	0	0	0	0
Pneumocyte Proliferation	0	0	2	2	3	2	0	0	0	0
Fibrinous Exudate / Hyaline Membrane	NO	NO	YES	YES	YES	YES	YES	YES	YES	YES
Alveolar Septal Edema	0	0	2	3	3	3	0	0	1	2
Alveolar Lymphocytes	0	0	1	1	1	1	1	1	1	1
Alveolar Eosinophils	0	0	1	1	2	1	1	1	1	1
Alveolar Neutrophils	0	0	1	2	1	2	1	1	0	1
Alveolar Histiocytes	0	0	2	3	3	2	1	1	1	2
Possible Viral Cytopathic Effect	NO	NO	YES	YES	YES	YES	NO	NO	NO	NO

**Fig. 9:**
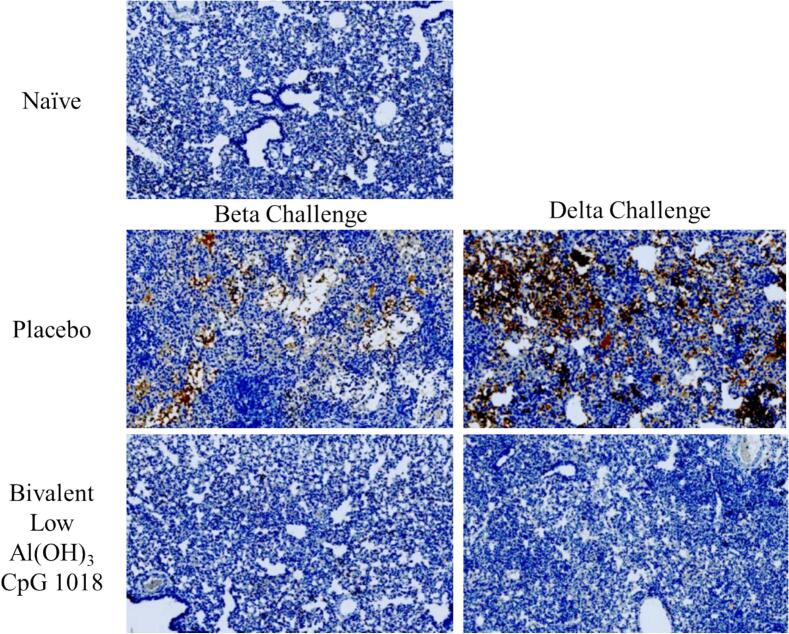
Immunohistochemistry of SARS-CoV-2 Nucleocapsid in lungs of vaccinated hamsters challenged with Beta or Delta SARS-CoV-2 virus. Immunohistochemistry on lung tissue harvested 5 days post-challenge. Anti-SARS-CoV-2 nucleocapsid antibody is displayed in brown. (For interpretation of the references to colour in this figure legend, the reader is referred to the web version of this article.)

Immunohistochemistry from lungs harvested 5-days post-challenge can be seen in Fig. 9. Distinct staining to the SARS-CoV-2 nucleocapsid can be seen in Placebo group, particularly the animals challenged with Delta. In contrast, hamsters that received Bivalent Low Al(OH)_3_ and CpG vaccine had no staining to SARS-CoV-2 nucleocapsid, comparable to the unvaccinated and unchallenged naïve group. This data is consistent with viral load data in Fig. 7.

Together this data shows that S-2P-rCRM197 conjugate vaccines with aluminum hydroxide with and without CpG 1018 induce robust immune responses and lead to protection from disease in animals.

## 4 Discussion

Demonstrated here are highly immunogenic protein-protein SARS-CoV-2 conjugate vaccines that induce nAb capable of inhibiting VOC that emerged long after the antigenic strains and effectively protect hamsters from disease. The vaccine candidates were protective when adjuvanted with aluminum hydroxide alone but addition of TLR agonists resulted in superior immunogenicity. Protein-based vaccine manufacturing is well-established and the resulting drug substances possess strong stability profiles. Long term stability studies were beyond the scope of this investigation; however, the drug substance in this study was shown to have a robust stability profile [12,14] establishing a good foundation for the resulting drug product.

mRNA vaccines can be produced quickly compared to protein-subunit vaccines, with Moderna's mRNA SARS-CoV-2 vaccine being administered to first patient in first-in-human trials within 10 weeks of the genetic sequence being available [47]. By utilizing the CHO^2353^ stable pool platform for antigen expression, GMP cell pooled banks can be produced and scaled up within 8-weeks [14]. Subsequent expression, downstream processes, and quality control can take another 8-weeks, meaning preclinical material would be available 16-weeks from sequence publication. Should regulatory guidelines be altered to allow pooled cells for early-phase clinical material, the time for protein-based vaccines to reach clinical phases would be greatly accelerated.

A major limitation of the study is the lack of cell mediated immunity data. Due to unforeseen circumstances, such studies could not be performed and are not feasible in the future. Nevertheless, published data demonstrates that CpG 1018 leads to strong Th1 cell mediated immunity [[48], [49], [50]]. Given this, it is expected that the formulations presented here would behave similarly.

We initially hypothesized that the Bivalent candidate would be superior, hence the accelerated path to challenge testing in hamsters. An additional shortcoming of the study was initiating the challenge study before obtaining all nAb data from mice, resulting in Beta-only conjugate vaccines not being tested in challenge studies. Nevertheless, the Bivalent vaccine exhibited robust results in the hamster challenge, with candidates containing CpG 1018 performing the best, confirming that the Beta – Delta conjugate vaccine with CpG 1018 could be suitable as a standalone vaccine.

The Beta-only High CpG 1018 candidate when tested in mice induced the highest serum IgG and nAb titers of all vaccine candidates and led to broad cross-protection against all variants tested, including Omicron BA.4 and Omicron BA.5 which emerged long after the Beta variant. Such broad cross protection to VOC was not observed in similar studies of monovalent spike-based vaccines [[51], [52], [53]], highlighting the quality of the neutralizing antibodies produced in response to this vaccine candidate [23,59,60] Although studies addressing long-term serological titers and memory immune responses were beyond the scope of this project, memory responses exhibited in other bacterial [22] and recently viral [54] conjugate vaccines suggests that these candidates could potentially induce similarly long-lasting protection from disease with the added benefit of broad cross protection to variants[56,57,61].

Common cold like viruses such as SARS-CoV-2, Influenza and Respiratory Syncytial Virus replicate in the respiratory mucosa, have relatively short incubation periods, do not cause marked viremia, and do not elicit long-term protective immunity from natural infection. Similarly, vaccines targeting these diseases generally do not provide durable protection or control of disease [55]. Frequent boosters with updated COVID-19 vaccines have been necessary to maintain immunity against evolving VOC. Continual vaccine modifications are financially burdensome and not ideal. The techniques employed in this study could be applied to the ongoing pursuit of universal vaccine to SARS-CoV-2 and other respiratory viruses that require additional modulations to boost their immunogenicity and breadth of coverage.

By adapting our bacterial conjugation techniques to protein subunit vaccines, and combining this with TLR agonist adjuvants we produced a vaccine with enhanced immunogenicity capable of broad cross-protection against VOC including those that were in circulation up to three years after the antigenic strains first appeared. These techniques can be pursued as potent mechanisms to enhance immunogenicity and breadth of protection offered by protein-based vaccines and shows great potential for viruses both current and yet to emerge.

## Credit authorship contribution statement

**Melanie Carroll:** Writing – review & editing, Writing – original draft, Methodology, Investigation, Formal analysis, Data curation, Conceptualization. **Heather B. Fox:** Writing – review & editing, Writing – original draft, Methodology, Investigation, Formal analysis, Data curation. **Anh Tran:** Writing – review & editing, Writing – original draft, Methodology, Investigation, Formal analysis, Data curation. **Gowri Chellappan:** Writing – review & editing, Methodology, Investigation, Formal analysis. **Leonardo V. Rojas:** Writing – review & editing, Investigation, Formal analysis. **Geetha Karengil:** Writing – review & editing, Writing – original draft, Methodology, Investigation. **Fataneh Karandish:** Writing – review & editing, Methodology, Investigation. **John W. Langston:** Writing – review & editing, Methodology, Investigation. **Brent M. Fall:** Writing – review & editing, Investigation. **Mary M. Whalen:** Writing – review & editing, Investigation. **Michael J. McCluskie:** Writing – review & editing, Methodology, Investigation. **Yves Durocher:** Methodology, Resources. **Anup Datta:** Writing – review & editing, Conceptualization. **Subhash V. Kapre:** Writing – review & editing, Funding acquisition, Conceptualization. **Ivan A. Olave:** Writing – review & editing, Supervision, Funding acquisition, Conceptualization.

## Declaration of competing interest

The authors declare the following financial interests/personal relationships which may be considered as potential competing interests:

Yves Durocher has patent #20230174591 issued to Yves Durocher. If there are other authors, they declare that they have no known competing financial interests or personal relationships that could have appeared to influence the work reported in this paper.

## Data Availability

Data will be made available on request.

## Appendix A Supplementary data

Supplementary material Image 1
