# Supplementary material for: SARS-CoV-2 conjugate vaccine elicits robust immune responses that can protect against evolving variants
Source: Vaccine. 2025 Apr 30;54:None. doi: 10.1016/j.vaccine.2025.126988 (PMC12132043; doi:10.1016/j.vaccine.2025.126988)
Supplement: Supplementary file 1 — Supplementary material [file mmc1.docx]

Supplemental Data


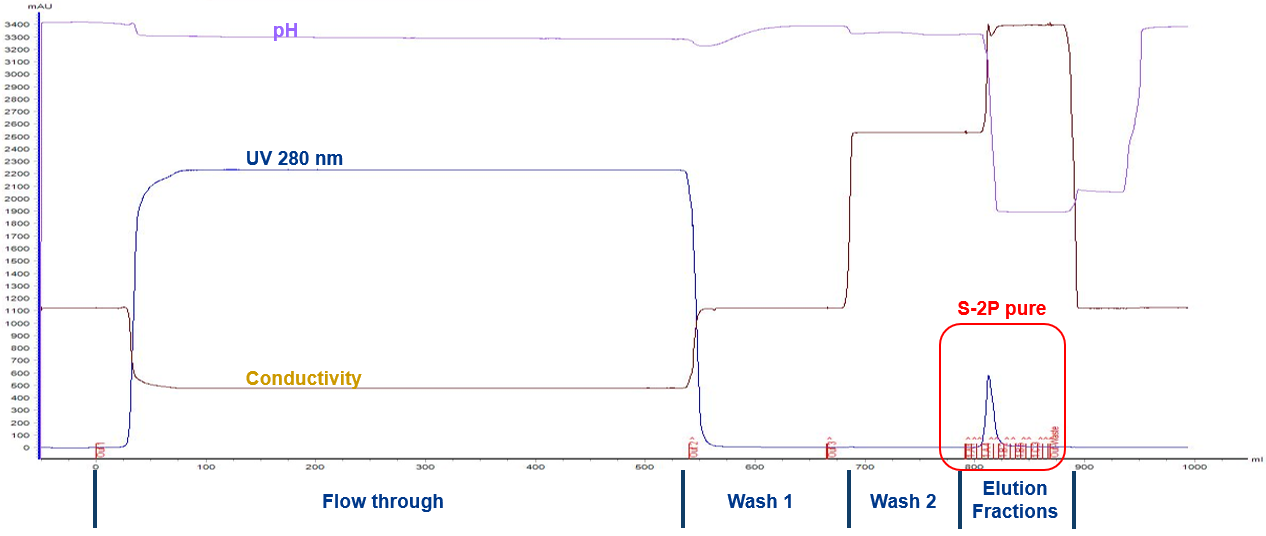


**Supplementary Figure 1:** **Representative S-2P Beta chromatography** data. 600 mL of thawed Beta S-2P protein (pH8) was loaded onto 12.5 mL of NGL Covid-19 Spike Protein AR 2.0 Affinity resin (Repligen) equilibrated with PBS, pH 7.4 using AKTA Avant 150 system (Cytiva Life Sciences) with a residence time of 4.9 minutes. Bound material was washed with PBS, pH 8.0 for 5 CV and 0.2 M NaCl, PBS, pH 8.0 for 5 CV. Protein was eluted using 3CV of 0.1 M sodium acetate, 1 M arginine, pH 5. Eluted, purified S-2P protein highlighted in red.


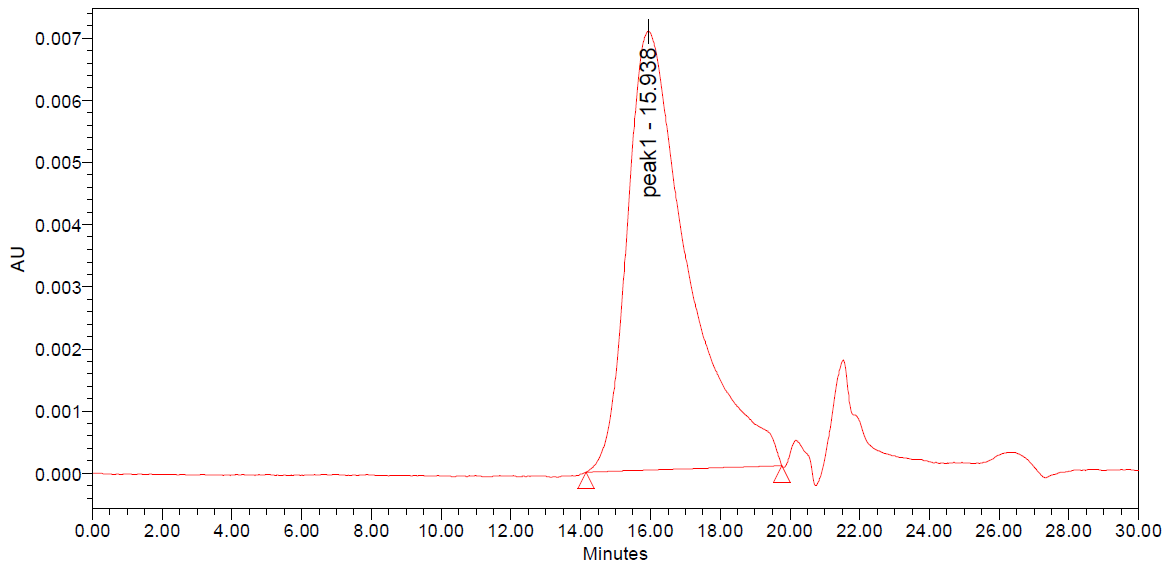


**Supplementary Figure 2:** **Representative SEC-HPLC profile of SARS-CoV-2 S-2P Delta protein** detected at 220 nm (red trace). Protein of interest has a retention time of 15.938 minutes and is labeled as peak1. Sample was run on Shodex 804 and 805 columns in series and eluted using 10 mM Potassium Phosphate buffer, pH 7.0 at 1.0 mL/min for a run time of 30 minutes. Buffer peaks at R_t_ > 21.00 min are excluded from the SEC-HPLC profiles.

1.
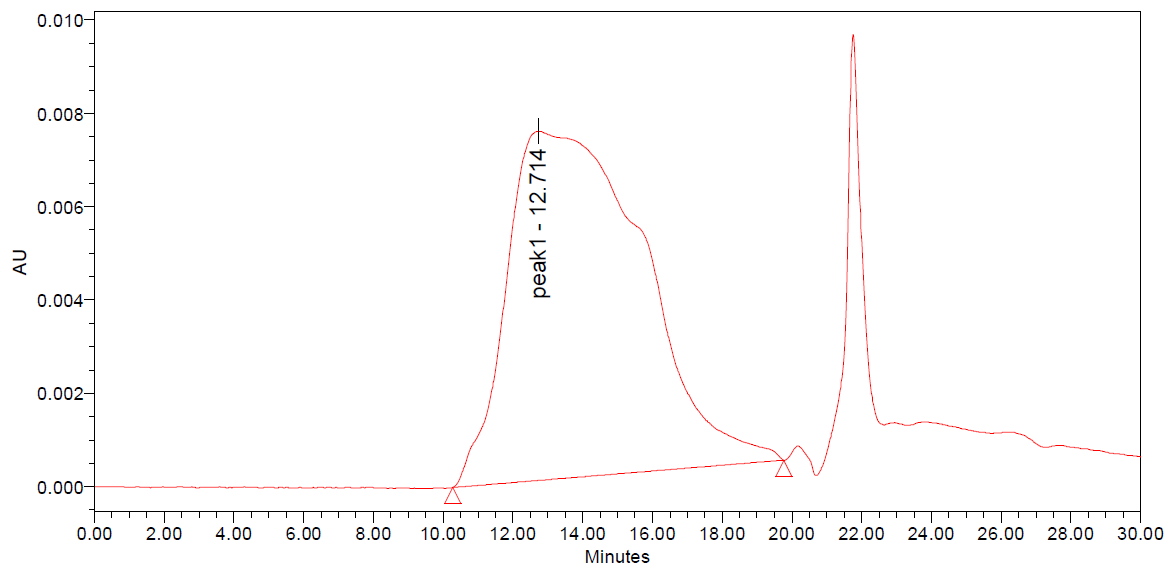

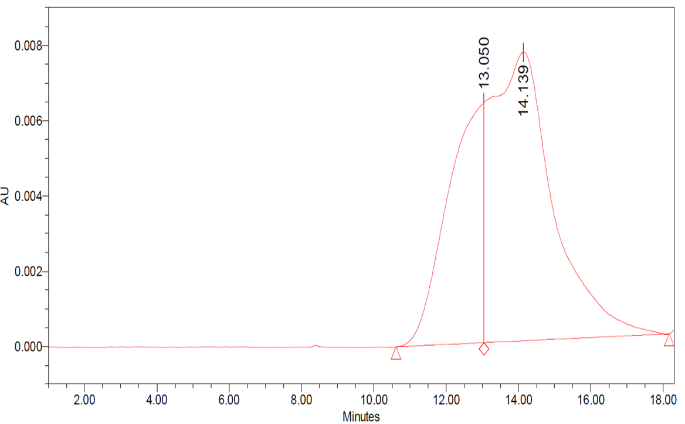
b)

**Supplementary Figure 3:** **Representative SEC-HPLC profiles of SARS-CoV-2 S-2P proteins-rCRM 197 linker conjugates** using **a)** Beta and **b)** Delta variants detected at 220 nm (red trace). Samples were run on Shodex 804 and 805 columns in series and eluted using 10 mM Potassium Phosphate buffer, pH 7.0 at 1.0 mL/min for a run time of 30 minutes. Buffer peaks at R_t_ > 18.00 or 20.00 min are excluded from the SEC-HPLC profiles.

| **Host Cell DNA** | **Host Cell Protein** |
| --- | --- |
| 0.088 ng / 5 µg dose | 0.015 ng / 5 µg dose |

**Supplementary Table 1:** Beta S-2P residual Host Cell DNA and Host Cell Protein

Nucleic acids were isolated with PrepSEQ Nucleic acid extraction kit (Thermo Fisher) and CHO Host cell DNA was detected using resDNASEQ Quantitative CHO DNA Kit (Thermo Fisher) as per manufacturer’s instructions. qRT-PCR was performed on Quantstudio 7 Pro (Applied Biosystems) and analyzed with Design and Analysis software 2.0. Host cell protein was detected using CHO Host Cell Proteins 3^rd^ generation immunoenzymetric assay for the measurement of CHO host cell proteins (Cygnus Technologies) as per manufacturer instructions.

| Beta/Delta S-2P variant (wt ratio) | CRM-PEG-COOH  (wt ratio) | EDC  (wt ratio) | s-NHS  (wt ratio) | COVID protein Activation pH | Beta/Delta protein-rCRM197-PEG-COOH Conjugation Reaction pH |
| --- | --- | --- | --- | --- | --- |
| 1 | 0.4 | 1.5 | 1.2 | 5.2±0.2 | 6.6±0.2 |

**Supplementary Table 2:** Reagent ratios and conditions used for conjugate preparation with Beta and Delta variants and rCRM197-PEG-COOH linker using Carbodiimide chemistry.
